# Tanshinone IIA Improves Ventricular Remodeling following Cardiac Infarction by Regulating miR-205-3p

**DOI:** 10.1155/2021/8740831

**Published:** 2021-11-29

**Authors:** Peng Qiao, Jie Xu, Xueni Liu, Xuehan Li

**Affiliations:** ^1^Department of Traditional Chinese Medicine, Yantaishan Hospital, Yantai, China; ^2^Department of Medical Security Center, PLA Rocket Force Characteristic Medical Center, Beijing, China; ^3^Critical Care Medicine, PLA Rocket Force Characteristic Medical Center, Beijing, China; ^4^Department of Geriatrics, Liaocheng People's Hospital, Liaocheng, China

## Abstract

**Objective:**

To illustrate the role of tanshinone IIA (TSN) in regulating cardiac structure and function following myocardial infarction (MI) and the involvement of miR-205-3p in TSN-induced antifibrosis effect on ventricular remodeling. *Patients and Methods*. One hundred MI patients were randomly assigned into two groups, and they were treated with TSN (TSN group, *n* = 50) or conventional therapy (control group, *n* = 50). Plasma levels of miR-205-3p and TGF-*β*1 were detected in each patient. Echocardiography was conducted in each patient at post-MI 1 day, 2 weeks, and 4 weeks, respectively, for recording LVIDd (left ventricular internal-diastolic diameter), LVIDs (left ventricular internal-systolic diameter), and LVEF (left ventricular ejection fraction). The interaction between miR-205-3p and TGF-*β*1 was examined by the RNA-Binding Protein Immunoprecipitation (RIP) assay. After induction of TGF-*β*1 and/or 10 *μ*L of TSN in cardiac fibroblasts, relative levels of miR-205-3p, Col1a1, and Col3a1 were detected by quantitative real-time polymerase chain reaction (qRT-PCR).

**Results:**

Compared with the control group, miR-205-3p and TGF-*β*1 were downregulated in plasma of MI patients in the TSN group. In the TSN group, LVIDd and LVIDs were reduced, and EF was enhanced at 2 weeks and 4 weeks compared with that at post-MI 1 day. miR-205-3p could negatively interact with TGF-*β*1. TSN induction abolished the regulatory effects of TGF-*β*1 on downregulating miR-205-3p and upregulating Col1a1 and Col3a1 in cardiac fibroblasts.

**Conclusions:**

Through upregulating miR-205-3p and downregulating TGF-*β*1, TSN alleviates cardiac fibrosis and improves ventricular remodeling following MI.

## 1. Introduction

The treatment of acute myocardial infarction (AMI) has been well concerned owing to its high incidence and mortality [1]. Due to the development of thrombolysis and interventional technology, the mortality of MI has sharply decreased. However, ventricular remodeling at post-MI should be worried. The process of ventricular processing involves multiple factors, including hypertrophy and apoptosis in cardiomyocytes and extracellular matrix (ECM) changes. Eventually, cardiac fibrosis results in heart failure, which directly affects the prognosis and life quality [1].

Tanshinone IIA (TSN) is the main liposoluble active component of Salvia miltiorrhiza (a traditional Chinese medicine) extracted from the dried root and rhizome of Salvia miltiorrhiza Bunge [2]. TSN is able to protect the myocardium and inhibit cardiomyocyte hypertrophy from cardiac injury [3]. Moreover, it suppresses cardiac fibroblast proliferation and collagen synthesis, thus exerting a vascular protection effect against fibrosis, improving coronary circulation and inhibiting thrombosis [4]. TSN has been extensively applied in the clinical treatment of vascular diseases, and its promising application has been reported in treating coronary disease, hypertension, cardiac ischemia-reperfusion injury, and arrhythmia [5].

miRNAs are endogenous, noncoding RNAs with 21-23 nucleotides long. They induce posttranscriptional regulation on target genes by complementary base pairing in the 3′-UTR [6]. As early as 2005, miRNAs relevant to cardiac functions have been reported [7]. With the gradual explorations on miRNA functions, people have realized that miRNAs are important in fibrotic diseases. It is reported that miR-29 participates in the development of cardiac fibrosis at post-MI *via* regulating cardiac fibroblast functions. miR-205 is located on chromosome 1q, which is abnormally expressed in many types of tumors and involved in regulating tumor cell behaviors [8, 9]. Downregulated miR-205 has been identified in breast cancer, prostate cancer, bladder cancer, and renal cancer samples [10–13]. miR-205 contains two subtypes from the same precursor, that is, miR-205-3p and miR-205-5p. miR-205-3p serves as a tumor suppressor in ovarian cancer, which is able to attenuate proliferative and migratory capacities of cancer cells [14]. The biological functions of miR-205-3p in MI and potential mechanism are rarely reported.

TGF-*β* is a family of cytokines with various functions. It regulates not only physiological processes, such as embryonic development, cell growth, and differentiation, but also pathological processes, such as inflammation, fibrosis, and carcinogenesis. TGF-*β* is a vital regulator in the development of tissue fibrosis [15]. This study mainly detected changes of fibrosis indicators following MI and the intervention effect of TSN. The potential involvement of miR-205-3p during TSN-induced improvement of ventricular remodeling was examined. Our results provide experimental references for clinical application of TSN in treating ventricular remodeling at post-MI.

## 2. Patients and Methods

### 2.1. Subjects

This study was approved by the Ethics Committee of Qingdao Central Hospital before the study started. Besides, signed written informed consent was obtained from all participants before the study. One hundred MI patients with primary acute ST elevation of the anterior wall who were treated by percutaneous coronary intervention (PCI) within 12 h of onset in Qingdao Central Hospital from May 2017 to May 2019 were recruited. Inclusion criteria were in accordance to *2013 ACCF/AHA guideline for the management of ST-elevation myocardial infarction* [16] as follows: (1) sudden ischemic chest pain symptoms, not relieved by rest or sublingual nitroglycerin; (2) ECG showing ST elevation (≥0.2 mV) of at least two adjacent precordial leads or left bundle branch block; (3) increase in CKI level more than twice than the normal one and/or troponin (+); (4) onset of AMI within 12 h; and (5) anterior descending artery lesion confirmed by coronary angiography and TIMI 3 graded after vascular opening. Exclusion criteria were as follows: (1) severe hepatorenal insufficiency, (2) severe valvular heart disease, (3) previous history of coronary heart disease, (4) cardiogenic shock, (5) history of major surgery or severe trauma in the past 15 days, (6) symptoms of cardiac insufficiency prior to AMI, (7) acute or chronic infection, (8) administration of NSAIDs or steroids, (9) autoimmune diseases, and (10) malignant tumors.

### 2.2. Grouping and Therapeutic Strategies

Patients were randomly classified into the control group (*n* = 50) and TSN group (*n* = 50). Emergency PCI was conducted in each patient, including medications of aspirin, clopidogrel, statins, ACEI or ARB, *β*-blockers, spironolactone tablets, and nitrates. In the TSN group, daily intravenous administration of 60-80 mg TSN for consecutive 14 days was additionally conducted.

### 2.3. Blood Collection

After overnight fasting, venous blood samples (3 mL × 2) were collected in the morning at day 1 and week 2 after admission. Samples were stored in ethylenediaminetetraacetic acid- (EDTA-) K2 anticoagulant tubes, centrifuged at 3000 rpm for 15 min and 12000 rpm for another 10 min. Plasma samples were placed in cryopreservation tubes, labeled, and stored at -80°C.

### 2.4. Echocardiography

Echocardiography was performed by an experienced ultrasound physician using the color Doppler ultrasound (Philips HD-15, S5-2 probe). At post-MI 1 day, 2 weeks, and 4 weeks, LVIDd (left ventricular internal-diastolic diameter), LVIDs (left ventricular internal-systolic diameter), and LVEF (left ventricular ejection fraction) were recorded as the average from three replicates.

### 2.5. Isolation of Rat Primary Cardiac Fibroblasts and Cell Culture

Rat primary cardiac fibroblasts were isolated and cultured as previously described [17]. Briefly, hearts of neonatal rats within 24 h were collected and washed in phosphate-buffered saline (PBS) at 4°C and 75% ethanol. Hearts were cut into small pieces and digested in 0.1% collagenase and 0.125% trypsin in a water bath at 37°C. The mixture was gently shaken every 10 min. Digestive solution was mixed in Dulbecco's modified Eagle medium (DMEM) (Gibco, Rockville, MD, USA) containing 10% fetal bovine serum (FBS) (Gibco, Rockville, MD, USA) and cultured in a culture bottle. The adherent time difference between cardiomyocytes and myofibroblasts was 60 minutes. Primary cardiac fibroblasts were regularly passaged using 0.25% trypsin, and the third generation was used for the following experiments.

### 2.6. TGF-*β*1 and/or TSN Induction

Cardiac fibroblasts were cultured in 6-well plates until 70-80% of confluence and then cultivated in serum-free DMEM for 24 h. TGF-*β*1 and/or 10 *μ*M TSN was applied per well for 24 h.

### 2.7. Cell Transfection

Cardiac fibroblasts were cultured in 6-well plates with 1 × 10^6^ cells per well in serum-free DMEM for 24 h. Until cell confluence reached 80-85%, transfection was conducted using Lipofectamine 2000 (Invitrogen, Carlsbad, CA, USA). 3 *μ*g miR-205-3p mimics or negative control and 6 *μ*g Lipofectamine 2000 were, respectively, suspended in 250 *μ*L of serum-free medium. After letting them stand at room temperature for 5 min, they were mixed and maintained for 20 min. The mixture was applied per well for 36-48 cell culture.

### 2.8. Quantitative Real-Time Polymerase Chain Reaction (qRT-PCR)

Total RNAs were isolated from plasma or cells using the RNA extraction kit (ABI, Applied Biosystems, Foster City, CA, USA). The concentration and purity of RNA were determined using an ultraviolet spectrophotometer (Thermo Fisher Scientific, Waltham, MA, USA). After reverse transcription, complementary deoxyribose nucleic acids (cDNAs) were amplified at 92°C for 2 min, followed by 35 cycles at 95°C for 20 s, 60°C for 40 s, and 72°C for 2 min. Relative mRNA level was calculated by 2^−ΔΔCt^. Primer sequences were as follows: miR-205-3p: 5′-CGG GAT TTC AGT GGA GTG AAG TTC-3′; TGF-*β*1: 5′-GCG CCT GCA GAG ATT CAA GTC AAC-3′ (forward) and 5′-GTA TCA GTG GGG GTC AGC AGC C-3′ (reverse); Col1a1: 5′-TTC ACC TAC AGC ACG CTT GT-3′ (forward) and 5′-TTG GGA TGG AGG GAG TTT AC-3′ (reverse); Col3a1: 5′-TTG AAT ATC AAA CCG CAA GGC-3′ (forward) and 5′-GGT CAC TTT CAC TGG TTG ACG A-3′ (reverse); and U6: 5′-CTC GCT TCG GCA GCA CA-3′ (forward) and 5′-AAC GCT TCA CGA ATT TGC GT-3′ (reverse).

### 2.9. RNA-Binding Protein Immunoprecipitation (RIP)

The Magna RIP Kit (Millipore, Billerica, MA, USA) was used. Cells were lysed in RIPA and incubated with magnetic beads conjugated with anti-TGF-*β*1 or anti-IgG at 4°C for 6 h. Subsequently, magnetic beads were washed and incubated with Proteinase K for clearing proteins. Purified RNAs were subjected to qRT-PCR.

### 2.10. Statistical Analysis

Statistical analyses were conducted using Statistical Product and Service Solutions (SPSS) 20.0 (IBM, Armonk, NY, USA). Measurement data between groups were compared using Student's *t*-test, while enumeration data were compared by the chi-square test. One-way ANOVA tests followed by the post hoc test (least significant difference) were employed for multigroup comparison. *p* < 0.05 was considered statistically significant.

## 3. Results

### 3.1. Baseline Characteristics between the Control and TSN Groups

We recruited 100 MI patients and randomly classified them into the control group (*n* = 50) and TSN group (*n* = 50). Their baseline characteristics were collected for comparison. It is shown that no significant differences in age, gender, hypertension, diabetes, smoking, LDL-C, HDL-C, and vascular opening time were found between groups (*p* > 0.05) (Table 1), suggesting that baseline characteristics were comparable.

### 3.2. Echocardiography Findings between the Control and TSN Groups

Echocardiography was conducted at post-MI 1 day, 2 weeks, and 4 weeks, respectively. On the first day following MI, no significant differences in LVIDd, LVIDs, and EF were found between the control and TSN groups (*p* > 0.05). These indicators were improved at post-MI 2 and 4 weeks in both groups, which were much more pronounced in the TSN group (Table 2). It is indicated that TSN treatment displayed a pronounced efficacy on improving cardiac structure and functions following MI.

### 3.3. Intervention of TSN on Plasma Levels of miR-205-3p and TGF-*β*1 in MI Patients

**Figure 1:**
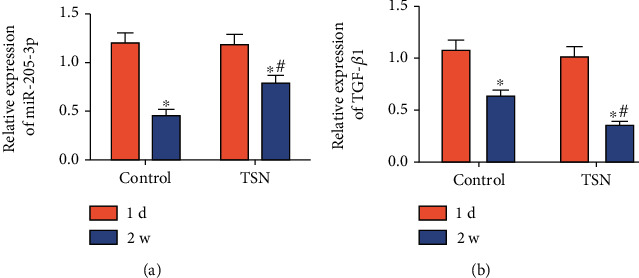
Intervention of TSN on plasma levels of miR-205-3p and TGF-*β*1 in MI patients. One hundred MI patients were randomly classified into the control group (*n* = 50) and TSN group (*n* = 50). Plasma levels of miR-205-3p (a) and TGF-*β*1 (b) were detected by qRT-PCR at post-MI 1 day and 2 weeks, respectively, in both groups.

We did not find significant differences in plasma level of miR-205-3p before treatment between groups. After two-week treatment, miR-205-3p was markedly downregulated in both groups, especially the TSN group (Figure 1(a)). Similarly, TGF-*β*1 was downregulated two weeks later after treatment in the control group, which is much more pronounced in the TSN group (Figure 1(b)). It is indicated that TSN could intervene plasma levels of miR-205-3p and TGF-*β*1 in MI patients.

### 3.4. Interaction between miR-205-3p and TGF-*β*1

**Figure 2:**
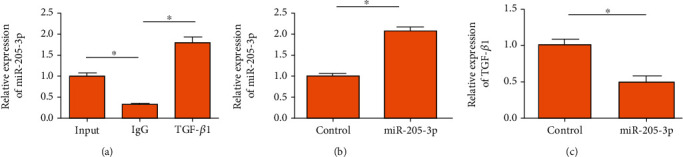
Interaction between miR-205-3p and TGF-*β*1: (a) RIP assay showed the interaction between miR-205-3p and TGF-*β*1; (b) transfection efficacy of miR-205-3p mimics in cardiac fibroblasts; (c) TGF-*β*1 level in cardiac fibroblasts overexpressing miR-205-3p.

The RIP assay revealed that miR-205-3p was mainly enriched in anti-TGF-*β*1, proving the interaction between miR-205-3p and TGF-*β*1 (Figure 2(a)). Subsequently, we tested the transfection efficacy of miR-205-3p mimics in cardiac fibroblasts (Figure 2(b)). Overexpression of miR-205-3p remarkably downregulated TGF-*β*1, showing a negative interaction (Figure 2(c)).

### 3.5. TSN Inhibited TGF-*β*1-Induced Cardiac Fibroblast Fibrosis by Upregulating miR-205-3p

**Figure 3:**
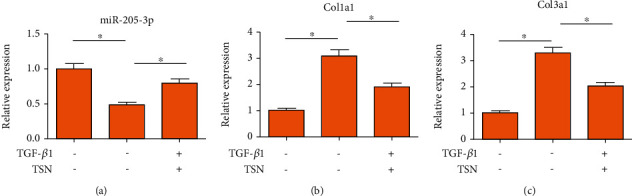
TSN inhibited TGF-*β*1-induced cardiac fibroblast fibrosis by upregulating miR-205-3p. Cardiac fibroblasts were induced with TGF-*β*1 and/or 10 *μ*L TSN. Relative levels of miR-205-3p (a), Col1a1 (b), and Col3a1 (c) were detected by qRT-PCR.

To explore the protective role of TSN in MI, cardiac fibroblasts were induced with TGF-*β*1 and/or 10 *μ*L TSN. TGF-*β*1 induction markedly downregulated miR-205-3p in cardiac fibroblasts, which was reversed by TSN treatment (Figure 3(a)). Moreover, Col1a1 and Col3a1 were upregulated in TGF-*β*1-induced cardiac fibroblasts, and their levels were reduced by TSN treatment (Figures 3(b), 3(c)). It is demonstrated that miR-205-3p exerted a vital function during the TSN-induced antifibrotic process by mediating the TGF-*β* signaling.

## 4. Discussion

At post-MI, ventricular remodeling results in changes of ventricular volume, ventricular shape, wall thickness, and compliance. The decline in cardiac function directly influences the prognosis in MI patients [18]. At present, drugs such as ACEI, ARB, and *β*-blockers are used for the treatment of AMI. They are conductive to inhibiting the process of myocardial fibrosis, improving ventricular remodeling, and facilitating the long-term prognosis of MI [19, 20]. However, to date, effective strategies for preventing ventricular remodeling at post-MI are lacking [21].

TSN is a fat-soluble active ingredient with clear molecular structure and molecular weight extracted from Chinese traditional medicine Salvia miltiorrhiza [21]. Ouyang et al. [4] suggested that TSN inhibits RAAS overrelease in the heart, thus decreasing the synthesis and release of collagens by cardiac fibroblasts to prevent cardiac fibrosis. Our results uncovered that 2-week or 4-week TSN treatment markedly improved cardiac functions in MI patients compared with echocardiography findings on the first day of admission. It is suggested that TSN can prevent ventricular dilation and contractility decline at post-MI, displaying certain significance in improving ventricular remodeling.

TGF-*β*1 exerts a key regulation in ECM metabolism [22]. It is recognized as the most important factor of fibrosis, which induces fibroblast proliferation and ECM accumulation through autocrine and paracrine [23]. Type I and III collagens are well studied. Type I collagen is featured by high tensile strength and poor stretch and retraction, which determines the rigidity of myocardial contraction and relaxation. Type III collagen has a good elasticity [24]. It is reported that increases in Col1a1 and Col3a1 lead to decreases in cardiac compliance, as well as systolic and diastolic functions [25, 26]. Accumulating evidences have highlighted the vital functions of miRNAs in ventricular remodeling at post-MI [27, 28]. In addition, the interaction between TGF-*β* and miRNAs is able to influence disease progression. Luna et al. [29] demonstrated that the negative interaction between TGF-*β*2 and miR-29b is responsible for regulating biological functions of trabecular cells of glaucoma. Qin et al. [30] suggested that miR-29b induces renal fibrosis progression through inactivating the TGF-*β*/Smad3 signaling.

Our results uncovered that both plasma levels of miR-205-3p and TGF-*β*1 were downregulated in MI patients. Furthermore, we have confirmed the negative interaction between miR-205-3p and TGF-*β*1. The upregulated Col1a1 and Col3a1 in cardiac fibroblasts induced by TGF-*β*1 were reversed by TSN treatment. To sum up, TSN upregulated miR-205-3p and thus inhibited TGF-*β*1 level, thereby improving cardiac function and alleviating cardiac fibrosis at post-MI. In this research, we uncover that TSN could improve ventricular remodeling in an in vivo and in vitro assay; however, the mechanism should be explored much more to perfect this hypothesis.

## 5. Conclusions

Through upregulating miR-205-3p and downregulating TGF-*β*1, TSN alleviates cardiac fibrosis and improves ventricular remodeling following MI.

## Figures and Tables

**Table 1:** Baseline characteristics of myocardial infarction patients in the control group and TSN group.

Variable	Control group (*n* = 50)	TSN group (*n* = 50)	*t*/*χ*^2^	*p*
Age	49.8 ± 5.9	48.2 ± 5.2	1.439	0.153
Sex (male/female)	37/13	34/16	0.437	0.66
TC (mmol/L)	5.66 ± 1.28	5.49 ± 1.13	0.704	0.483
LDL-C (mmol/L)	3.28 ± 0.95	3.37 ± 0.97	0.469	0.64
HDL-C (mmol/L)	1.08 ± 0.22	1.15 ± 0.24	1.52	0.132
Hypertension (*n*, %)	17	20	0.386	0.679
Diabetes (*n*, %)	11	8	0.585	0.611
Smoking (*n*, %)	20	23	0.367	0.686
Vascular opening time (h)	6.92 ± 1.26	7.17 ± 1.33	0.965	0.337

TSN: tanshinone IIA; TC: total cholesterol; LDL-C: low-density lipoprotein cholesterol; HDL-C: high-density lipoprotein cholesterol.

**Table 2:** Echocardiography findings in the control and TSN groups.

Variable	Control group (*n* = 50)			TSN group (*n* = 50)		
	1 d	2 w	4 w	1 d	2 w	4 w
LVIDd	51.52 ± 5.51	50.35 ± 4.98	50.27 ± 5.07	51.63 ± 6.08	47.52 ± 5.85^∗^^&^	47.66 ± 3.74^#&^
LVIDs	34.82 ± 4.21	32.82 ± 4.67	32.94 ± 4.55	34.95 ± 4.38	29.85 ± 3.17^∗^^&^	29.06 ± 2.97^#&^
EF (%)	42.33 ± 4.07	45.12 ± 5.51	45.97 ± 5.58^∗^	41.82 ± 4.13	48.72 ± 4.82^∗^^&^	49.57 ± 5.03^#&^

^∗^ *p* < 0.05, comparison between 1 d and 2 w; ^#^*p* < 0.05, comparison between 1 d and 4 w; ^&^*p* < 0.05, comparison between the control group and TSN group.

## Data Availability

The datasets used and analyzed during the current study are available from the corresponding author on reasonable request.
